# Supplementary material for: Bioaccessibility of Betalains in Beetroot (Beta vulgaris L.) Juice under Different High-Pressure Techniques
Source: Molecules. 2022 Oct 20;27(20):7093. doi: 10.3390/molecules27207093 (PMC9611175; doi:10.3390/molecules27207093)
Supplement: Supplementary file 1 [file molecules-27-07093-s001.zip › molecules-1934257-supplementary.pdf]

Supplementary material

Bioaccessibility of Betalains in Beetroot (*Beta vulgaris L.)* Juice under different High-Pressure Techniques

Urszula Trych[1,*], Magdalena Buniowska-Olejnik[2] and Krystian Marszałek[1,3*]

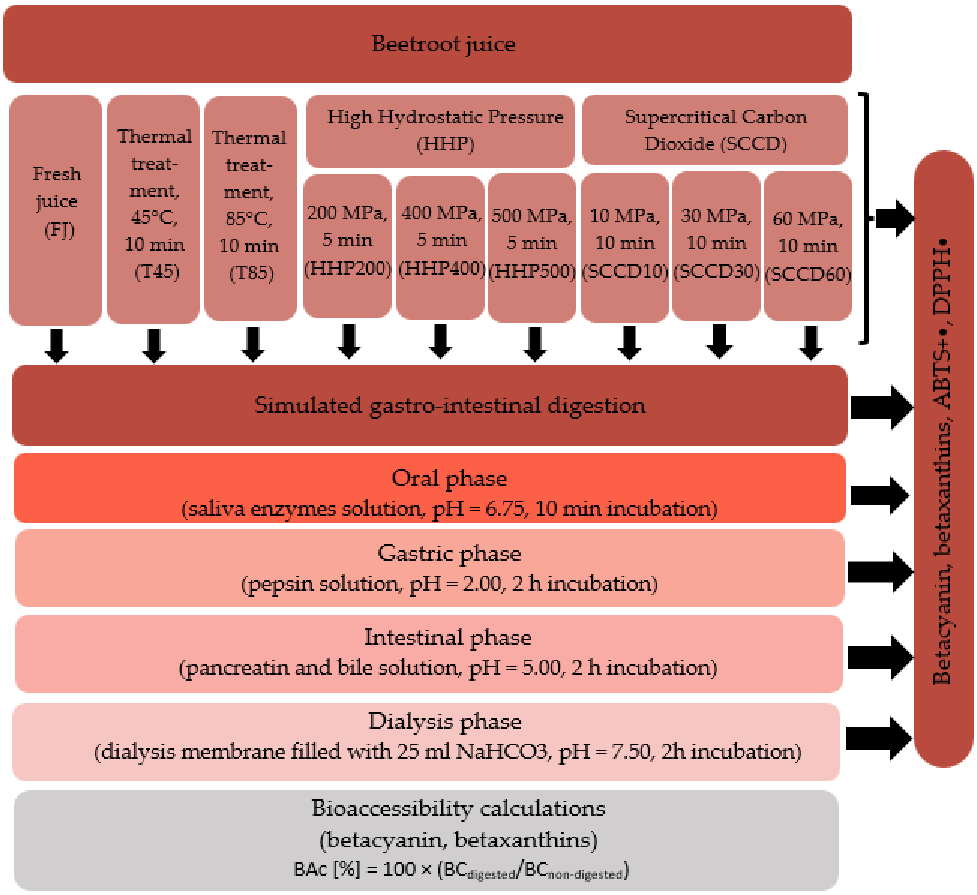

Figure S1. The scope and the workflow of the research

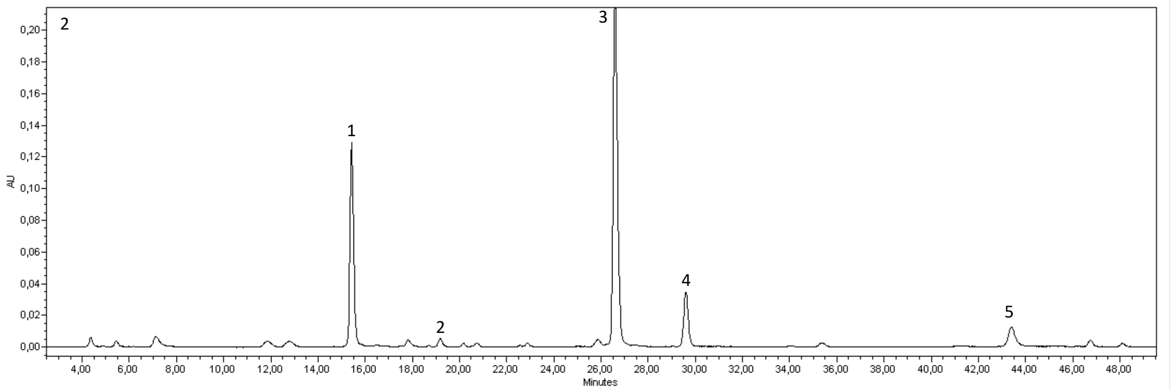

Figure S2. Chromatogram showing HPLC analysis of betaxanthins in fresh beetroot juice (FJ) at 480 nm. 1- vulgaxanthin I (RT= 15 min), 2- vulgaxanthin II (RT= 19min), 3- betanin (RT=27 min), 4- isobetanin

(RT=29 min), 5- neobetanin (RT=43 min), other small peaks from 4 to 26 min was quantified together and described as "other betaxanthins".

Betanin and isobetanin belongs to the betacyanins group and they were quantified at 538 nm due to much bigger peaks occurred at this wave length. Neobetanin is also one of the betacyanins, but its content was calculated at 480 nm due to stronger, more visible signal.

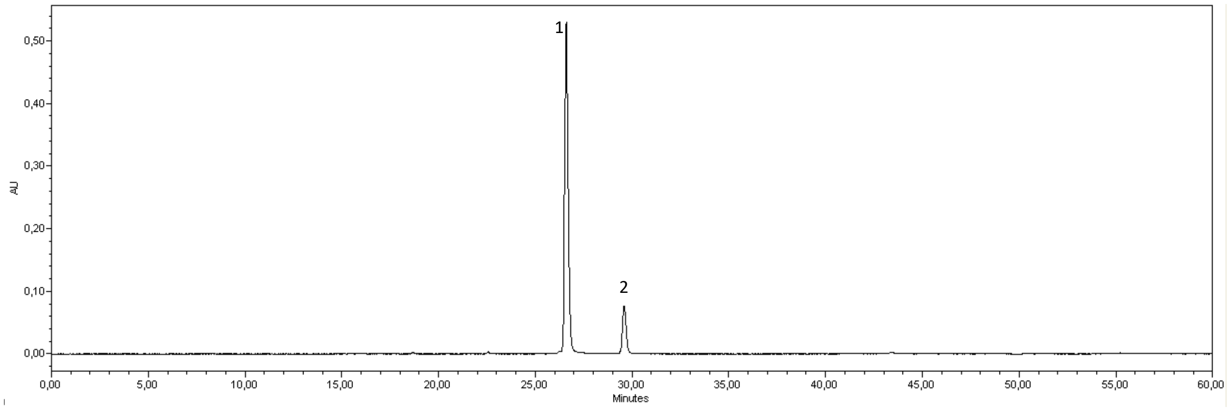

Figure S3. Chromatogram showing HPLC analysis of betacyanins in fresh beetroot juice (FJ) at 538 nm. 1-betanin (RT=27 min), 2- isobetanin (RT-29 min).
